# Monitoring Claw Length, Feet Infrared Temperature, Mobility and Backfat Tissue Changes in Replacement Gilts of Different Genetic Lines in Three Farrow-to-Finish Herds in Greece

**DOI:** 10.3390/vetsci10030199

**Published:** 2023-03-06

**Authors:** Fotios G. Kroustallas, Georgios A. Papadopoulos, Vasilis Skampardonis, Leonidas Leontides, Paschalis Fortomaris

**Affiliations:** 1 Laboratory of Animal Husbandry, Faculty of Veterinary Medicine, School of Health Sciences, Aristotle University of Thessaloniki, 54124 Thessaloniki, Greece; 2 Department of Epidemiology, Biostatistics and Economics of Animal Production, School of Veterinary Medicine, University of Thessaly, 43132 Karditsa, Greece

**Keywords:** infrared thermography, replacement gilts, claw length, anisodactylia, mobility, reproductive performance, backfat

## Abstract

**Simple Summary:**

Claw health condition is of major importance for modern hyperprolific sow productivity and longevity and pig operations profitability. Infrared technologies (IRTs) have been used in human medicine and recently in the claw health and foot temperature distribution evaluation of purebred sows. The current study presents the results of monitoring claw length, claw lesion, anisodactylia, backfat thickness, mobility and reproductive performance evolution from weaning age to second parturition in replacement gilts of different genetic lines (PIC, DANBRED and TOPIGS) in three farrow-to-finish herds in Greece, concurrently with temperature distribution evaluation using infrared technologies. The majority of the assessed characteristics showed statistical differences across herds at the first and second parturition, as well as at the weaning age. The fact that the three herds’ housing, flooring, management and feeding procedures were essentially the same suggests that there may be genetic variation in the analyzed parameters’ conformation. However, more research is necessary to clarify the relationships between the temperature distribution of the feet and the claw health indicators, mobility, backfat thickness and reproductive efficiency. The development of infrared technology as a practical, accurate and dependable technique for the early diagnosis of claw health issues in replacement gilts may be crucial for practicing veterinarians.

**Abstract:**

Feet infrared temperature is associated with feet health and may affect the reproductive performance of sows. In total, 137, 98 and 114 replacement gilts were selected at the age of weaning from 3 herds—A, B and C—with different genetic lines. Dorsal claw length was measured, and anisodactylia was measured in all four feet, at weaning age, and at those gilts that completed their first and second farrowing. At the first and second farrowing stage, the infrared temperature distribution, dew/claw length and backfat thickness were measured concurrently with claw lesion and mobility score evaluation. The maximum temperature significantly differed (*p* < 0.01) among herds, in the rear feet and in all four feet at the first and second farrowing respectively. Claw lengths statistically differed among herds at all stages (*p* < 0.05). Anisodactylia in rear feet was lower in herd A (*p* < 0.05) at weaning, and in herd C at the first and second farrowing (*p* < 0.05). In addition, the claw lesion score, mobility, backfat thickness and reproductive performance statistically differed among herds (*p* < 0.05). It is shown that even at an early stage of their reproductive life, claw length differences exist in replacement gilts of different genetic lines.

## 1. Introduction

Claw health in modern hyperprolific sows is important for their longevity, productivity and welfare. It is well recognized that sows with lameness experience pain and that their well-being is compromised. One of the primary reasons of sow lameness is the presence and severity of claw lesions [1]. Inconsistencies in the hoof formation, such as overgrowth and differences in the length of the medial and lateral claws, have been linked to lameness and claw abnormalities [2,3]. Claw length disparities have been associated with an increase in claw lesions frequency [4]. Claw lesion estimation is a labor- and time-intensive process, and clinical lameness can only be visually observed when a major claw lesion has occurred. The pig business might benefit from a method for the early diagnosis of affected damaged tissue in feet [5]. As a helpful non-invasive technology, infrared thermography (IRT) has been widely employed in human and veterinary medicine for diagnostic purposes [6,7,8,9]. Changes brought on by the progression of inflammation in the underlying tissues can be quickly identified using IR technology [10]. Other studies have connected changes in surface temperature to increased blood flow to tissues during inflammatory conditions [10,11,12]. The use of IRT to recognize lameness in livestock has increased largely in the last years since it represents a non-invasive method, ease of automation and low cost [13,14].

In sows, claw lesions are common, and their evaluation has been under debate in recent years. A proposed categorization system for claw lesions has been employed in the past [15]. It is based on a rating scale and includes fractures in the wall and toe, white line and heel–sole junction disruptions, heel curtailments and overgrowth. Others have used a scale with three or four [16] criteria to evaluate the length of the claw and dew claw, as well as lesions. More recently, a scoring system applied was “Zeugenklauwencheck” [16] and the Zinpro Foot First method [17].

It can be hypothesized that sows with abnormalities in claw length concurrently with increased incidence of claw lesions, would affect their ability to move as a result. Claw overgrowth and lesions have been linked to mobility difficulties in sows [2,18], as have weight distribution changes to the heel in cases of enlarged nails, which makes walking difficult [19]. Overgrowth of weight-bearing claws can affect sows’ lying down, postural and feeding behavior, with a negative impact on sows’ welfare and productivity [20]. Thus far, comparison of mobility issues together with claw length abnormalities and claw lesions has not been extensively evaluated in gilts of different genetic backgrounds. As mobility and claw health issues are a common reason for sow cull from a herd, it would be worthwhile to investigate whether such issues exist and persist from the beginning of the reproductive life of replacement gilts and whether differences exist among genetic lines. Furthermore, it could be hypothesized that gilts with claw abnormalities would have impaired mobility, either at the gestation group pens or at the lactation crates, which might subsequently affect their reproductive performance. Under such conditions, it is plausible that a gilt or a sow might be reluctant to frequently stand up to consume feed. Thus, during the high-metabolic-demand period of lactation, increased backfat tissue mobilization might occur.

Therefore, the current study’s aim was to further examine, using IRT devices, the temperature gradient in the lower front and rear feet of replacement gilts, while also monitoring the hypertrophy of their claws, the evolution of anisodactylia, the occurrence of claw lesions, the depth of their backfat, their mobility and their reproductive efficiency.

## 2. Materials and Methods

### 2.1. Herds and Gilts Included in the Study

The recent investigation with purebred sows that took part in the same research effort was done in the same herds as the current study [21]. In short, herds were situated in the Prefecture of Larisa (Herd A and C) and Kozani (Herd B), and the purebred breeding sows belonged to the PIC, TOPIGS and DANBRED genetic lines, respectively. Further, the housing, flooring and managerial practices at the insemination, lactation and gestation facilities were almost similar among herds, as described in detail in the aforementioned study. At the age of weaning, 349 crossbred replacement gilts were randomly selected, including 137, 98 and 114 (in herds A, B and C, respectively). Among the replacement gilts, 132, 84 and 110 finished their first farrowing (in herds A, B and C, respectively). In herds A, B, and C, there were 55, 68 and 47 individuals that finished the second farrowing.

### 2.2. Housing and Management

Housing and management at the insemination, gestation and lactation facilities are described in detail in the aforementioned study [21].

### 2.3. IRT Measurements

**Figure 1:**
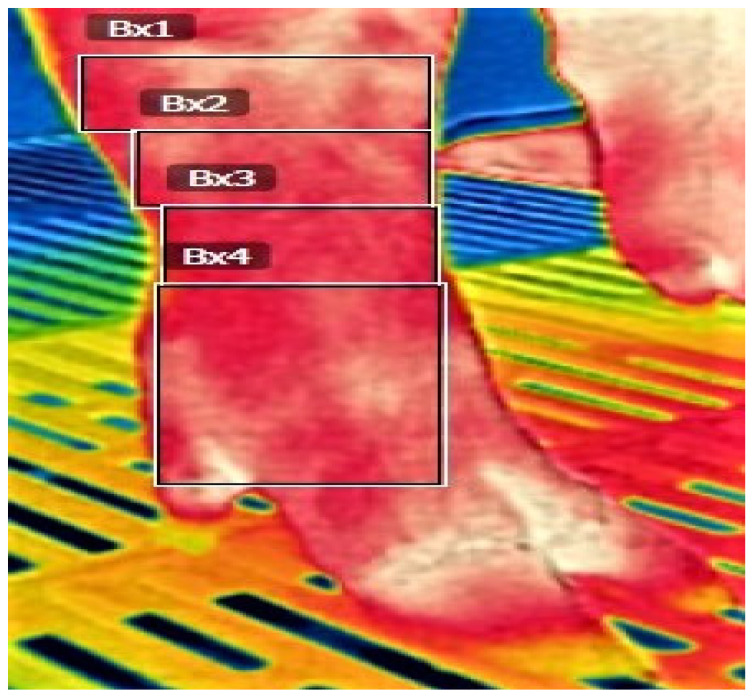
Areas of temperature measurement by IR camera in sows of different genetic lines in three farrow-to-finish herds in Greece: IRT1(Bx1) Carpus-Tarsus, IRT2(Bx2) Upper metacarpi/metatarsi, IRT3(Bx3) Lower metacarpi/metatarsi, IRT4(Bx4) Phalanges.

We followed the measurement procedure as previously described in our recent publication on purebred GP sows [21]. The areas of interest in each foot are shown in Figure 1.

### 2.4. Claw Length Measurements and Anisodactylia

At weaning day, the claw lengths of all female piglets selected as replacement gilts were measured, and all relevant information was recorded on forms together with an ID number and the day of the birth. In total, 137 female piglets from herd A, 98 from herd B and 114 from herd C were included. The claw length measurements were performed also after farrowing in those gilts that completed their first and second farrowing. In this manuscript, we present the results only for dorsal claw length.

### 2.5. Claw Lesion Scoring

**Figure 2:**
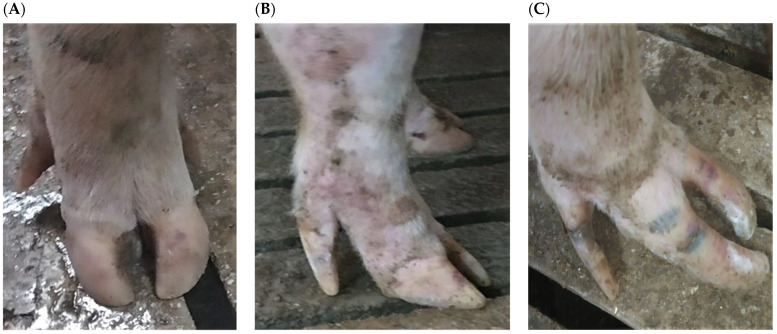
Examples of lesion scoring in sows of different genetic lines in three farrow-to-finish herds in Greece: (**A**) Claw: 0; Wall: 0; Coronary band: 0; Dew claw: 0. (**B**) Claw: 1; Wall: 1; Coronary band: 0; Dew claw: 1. (**C**) Claw: 2; Wall: 2; Coronary band: 1; Dew claw: 2.

When gilts were in the standing-up posture in the crates during the final week of lactation both at the first and second farrowing, the medial and lateral toes of every hoof and the dew claws were separately evaluated for lesions and graded. Using three classification scales with ratings varying from 0 to 2, a modified scoring method was utilized, based on previous studies [2,16,17]; see Table 1 and Figure 2.

### 2.6. Back Fat Measurements

Backfat tissue length was measured at the P2 position (6.5 cm from the backbone, left and right side) with a portable ultrasound device (Renco, Minneapolis, MN, USA). Gilts were measured at insemination, while the sow entered the farrowing pen, and on the weaning day.

### 2.7. Mobility Scoring

Sow lameness was estimated after the completion of the lactation period. Sows were video recorded for 3–4 min, using a portable digital camera (Sony, HDR-CX240E, Handycam, Tokyo), while they walked on the concrete floor in an alley of 5–10 m and on the slatted floor of gestation pens. A person would walk beside the sows and give verbal cues or wave his hands to get them moving if necessary. Their degree of lameness was scored at a later time. Sows were provided with a score of 1 (no lame) to 4 (unable to move), using an observation movement scoring system on a 4-point scale modified from Gregoire at al. [22]:

Score 1: The sow moves with even steps, is easy to move around, feels comfortable on all four feet and requires nothing in the way of incentive.

Score 2: Uneven gait length, stiffness and fewer smooth motions are noticed, but no observable lameness is evident.

Score 3: Shortened stride, one leg is found to be lame, the caudal body swaggers and compensatory actions are all observed in the sow as she walks (head drooping, back raised)

Score 4: More than one leg is lame, the sow is hesitant to put weight on the limbs that are afflicted, she does not rest the limb on the ground, and she is hesitant and unable to move.

### 2.8. Reproductive Performance

Reproduction data of each herd participating in the study were kept for all reproductive parameters, such as insemination date, expected farrowing date, farrowing date, total born piglets, born alive piglets, fostering between sows, lactation length, weaned piglets and age at the first farrowing.

### 2.9. Statistical Analysis

The Statistical Package for the Social Sciences (SPSS) program was used to examine the data (SPSS 25.0 Version, Chicago, IL, USA). The threshold for statistical significance was set at *p* = 0.05. With herds acting as a fixed factor in the one-way analysis of variance of the general linear model procedure of SPSS, differences among herds in terms of IRT, claw length, claw lesion, anisodactylia, backfat thickness and reproductive performance measurements were studied. Kolmogorov–Smirnov and Shapiro–Wilk tests were performed in order to evaluate the normal distribution of data, and post-hoc assessments among herds were then done using Tukey’s test. The data are displayed as mean standard deviation (SD).

## 3. Results

### 3.1. IRT Recordings

Table 2 displays the highest temperatures recorded during IRT recordings (mean SD) in the four lower-foot body sites of each sow at the first farrowing. The maximum temperature in IRT1, IRT2, IRT3 and IRT4 of almost all rear feet, with the exception of IRT4 in the rear right foot, were lower in herd A and higher in herd B (*p* < 0.001) at the first farrowing. All recorded temperatures values, in all three herds, at the first farrowing were numerically higher in rear feet than in the front.

Maximum IRT temperatures in both the front and rear feet for all regions estimated (IRT1, IRT2, IRT3 and IRT4) were different among herds, being the lowest in herd B compared to A and C (*p* < 0.001) Table 3. All recorded temperature values, in all three herds, at the second farrowing were numerically higher in rear feet than in the front.

### 3.2. Claw Length Measurements and Anisodactylia

The mean values (±SD) of the dorsal claw lengths of the medial and lateral claw of the front and rear feet at the weaning stage and at the first and second farrowing are shown in Table 4. At weaning, the dorsal claw lengths in all four feet statistically were lower in herd B than in A and C (*p* < 0.001). At the first farrowing, the claw lengths in both the front and rear feet were lower in herd C compared to A and B (*p* < 0.001). At the second farrowing, the dorsal claw lengths were also lower in herd C compared to A and B (*p* < 0.001).

In Table 5, the results regarding anisodactylia in each one of the four feet of the replacement gilts on the weaning day and at the first and second farrowing in the three herds are presented. Anisodactylia was lower in herd A at weaning than in herds B and C (*p* < 0.001). At the first farrowing, herd B had higher front feet than herds A and C (*p* < 0.001), and herd A had higher rear feet than herds B and C (*p* < 0.001). At the second farrowing, anisodactylia in the front left foot and both rear feet was higher in herd A (*p* < 0.001).

### 3.3. Claw Lesion Score

The total claw lesion score in each foot, the count of lesion scoring in the region of interest (claw, wall, coronary band and dew claw) in all four feet, was the highest in herd A (*p* < 0.001) at the first farrowing. In addition, at both feet, the medial and lateral claw lesions were significantly higher in herd A compared to B and C (*p* < 0.001). At the second farrowing, the total feet lesion score was the highest in herd A (*p* < 0.001), and for the front-rear feet, the medial-lateral claw lesion scores were greater in herd B compared to A and C (*p* < 0.001) Table 6.

### 3.4. Backfat Measurement and Backfat Loss

Table 7 presents the backfat thickness at the farrowing and weaning day, as well as the backfat loss and the backfat loss % at the first and second farrowing of sows. At the first farrowing, the backfat thickness, backfat loss and backfat loss % significantly differ among herds, being higher in herd B than in herd A and C (*p* < 0.001). At the second farrowing, herd B is the herd with the statistically highest values for all measured backfat parameters among all three herds (*p* < 0.05).

### 3.5. Mobility Score

The mobility score of sows was analyzed as the mean score of the three assessment scores. Mobility scoring was higher in herd A compared to B and C (*p* < 0.001 in the first farrowing; *p* = 0.002 in the second farrowing) Table 8.

### 3.6. Reproductive Performance

Table 9 shows the total born, born alive and weaned piglets for the first and second farrowing. At the first farrowing, the litter size parameters were greater in herd B than in A and C (*p* < 0.001). At the second farrowing, the reproductive parameters differed among the three herds, with herd B showing the best performance indexes (*p* < 0.001).

## 4. Discussion

The annual replacement rate of the sow reproduction population within each herd may reach up to 40%, originating from replacement gilts. In our previous investigation [21], we have shown that significant differences occurred among purebred sows of different genetic lines regarding the claw lengths, lower feet infrared temperature distribution and occurrence of anisodactylia. Based on these findings, we investigated the hypothesis if the replacement gilts originating from the same purebred sows would show similar claw abnormalities, starting from their selection at weaning age up to the completion of their second farrowing. The results showed that those gilts exhibiting claw length differences and higher lesion scores were prone to greater mobility issues. Meanwhile, reproductive performance indexes, such as backfat tissue mobilization during lactation and litter size at birth and at weaning, were collected. To our knowledge, this is the first study evaluating the claw condition in replacements and its effects on reproductive performance in the first two reproductive cycles. The results indicate that selection of a genetic line should not be focused only on litter size characteristics, but also on claw health condition.

In our investigation, other anatomical areas besides phalanges may be more vascularized, and hence, IRT may be able to show changes in the local metabolic rate and blood flow more clearly [18,23]. There was a substantial difference among the analyzed genetic lines in both the first and second farrowing, according to the measured IRT temperature distribution in the replacement gilts from three distinct herds. The IRT distribution in gilts at the first and second farrowing were in accordance with our findings in purebred sows’ feet temperature evaluation in our recent study [21]. It should be highlighted that in the four sites of focus, sows’ highest IRT temperatures were numerically higher in the posterior than the front feet (IRT1, IRT2, IRT3 and IRT4), which is consistent with previous reports from purebred sows [21]. It is possible that variations in the distribution of weight bearing are to blame for the greater IRT temperatures found in sows’ rear feet as opposed to their front feet. The formation of claw lesions in various feet in sows is greatly influenced by their weight distribution [24,25]. Interestingly, the rear feet lesions score was numerically higher than the front feet lesions score in the first and second farrowing. Thus, it can be suggested that differences in weight distribution exist even from the first farrowing in the replacement gilts, and differences in lesion scores are evident among different genetic lines.

Our findings for claw lengths at weaning are the first published data showing a range of mean values for claw lengths between 10.43 mm and 26.89 mm for front feet and 9.59 mm and 27.27 mm for rear feet. At the first and second farrowing, the lowest claw lengths were observed in herd C compared to A and B. Fick (2014) [26] reported sow lateral and medial dorsal claw length measurements that were shorter than ours. In terms of anisodactylia, there were also numerical differences in favor of the rear foot. This is consistent with earlier discoveries [27]. In contrast to our findings on the second farrowing, for the rear legs, Newman et al. [28] showed that enlarged and normal claw lengths ranged from 51 mm to 79 mm and 38 mm to 50 mm, respectively. Sasaki et al. [4] reported toe size measurements for the medial and lateral claws that ranged from 45.0 mm to 45.1 mm and 46.20 mm to 46.60 mm, respectively, in research that only examined the rear feet of gestating sows. These results are different from our findings at the first (55.16 mm and 58.10 mm for the medial and lateral rear feet claw, respectively) and second farrowing (62.92 mm and 66.52 mm for the medial and lateral rear feet claw, respectively). Sows with a toe length higher than 63 mm were categorized as having overgrowth in a study by Fitzgerald et al. [29]. Our data suggest that gilts from herds B and C, particularly when compared to those from herd A, have enlarged claws.

According to recent studies, the average increase of claws ranged from 0.21 mm/day for gilts to 0.39 mm/day for sows [30,31], and while the wear rate decreased to 0.17 mm/day, the inevitable claw length increased steadily and got shorter with age [31]. When compared to Yorkshire sows, the lateral toes of Duroc and Crossbred (Duroc Yorkshire) sows grow more quickly; the dorsal toes of sows at parity two grow the fastest, followed by those at parity one and three [31]. In our study, the mean growth rate (growth minus wear) for dorsal claw length was about 0.114 mm/day, 0.109 mm/day and 0.096 mm/day for herd A, B and C, respectively, slightly different to the findings of the latter studies for the first (weaning to first farrowing) and second (first to second farrowing) evaluated periods. These results may be indicative of a genetic influence in claw horn growth, which warrants further investigation.

Claws that are too long may cause sows to dedicate less time to eating [29]. It is known that feed consumption should be maintained at high levels during lactation to avoid excessive body weight and backfat loss due to increased milk production. It has been described that lame sows may have impaired body condition compared to non-lame ones consuming the same amount of feed [32]. Furthermore, cytokines derived from inflammatory procedures may not only be involved in connective tissue degradation, which worsens lameness, but may also influence the action of hormones involved in reproduction [32]. Significant inflammation and epithelial alterations may be linked to claw overgrowth [27,28]. Claw lesions have also been linked to poor litter performance [3,29]. As revealed by our findings, those gilts that showed higher backfat tissue loss during both lactation periods originated from herd B. The same gilts showed a higher lesion score at the rear and lateral claws at the second farrowing, but did not exhibit higher anisodactylia or IRT measurements compared to those from herds A and C. It should be noted that gilts from herd B had the best piglet performance indexes. Thus, it could be hypothesized that gilts may mobilize backfat tissue during lactation to support litter growth independently of claw length differences or overall feet condition. Nevertheless, this field warrants further investigation analyzing the relationships among the aforementioned parameters.

Lameness prevalence in all three herds at the first farrowing were 18.9% of total gilts and at the second, 48%. To estimate the prevalence of lameness, gilts with a locomotor value of greater than two were labeled as lame. The gilts originating from herd A had simultaneously greater mobility problems, a greater total lesion score and anisodactylia at both the first and second farrowing. It can be suggested that gilts with overgrown claws may be more prone compromised mobility, even at the stage of the first farrowing. This phenomenon remains persistent in the completion of the second farrowing. Further research is required due to the nature of the interactions among these parameters.

The housing, management practices and environmental factors were similar among the participating herds. In brief, the floor type was similar, weaning took place in all farms at 28 days, the gilts and sows were introduced to the gestation pen grouping at approximately 30 days after weaning, and the gestation pen groups were static and not dynamic, while the space allowance in the gestation pens was similar on all farms. According to Hallowel and Pierdon [33], such housing and environmental approaches may be connected to sow lameness. The usage of dynamic groups was suggested to increase the likelihood of lameness, along with increasing sow density in the latter trial. Overall, their influence on the analyzed parameters may not have been significant due to the three farms’ similar housing and management techniques. Yet, more research has to be done in this area.

## 5. Conclusions

Monitoring claw health and reproductive performance evolution from weaning age to the second parturition of replacement individuals is the main topic of the study. Our findings show that the IRT temperature pattern of lower feet varies, as well as the claw lengths, anisodactylia, mobility pattern and reproductive performance within replacement gilts of different genetic lines reared under almost similar housing, flooring conditions and management practices. Based on the results, it appears that gilts prioritize backfat tissue mobilization to support litter growth during lactation, independently of the extent of claw abnormalities. Still, those gilts with compromised mobility at the first and second farrowing were those with a great anisodactylia incidence and higher IRT measurements and claw lesion scoring. Yet, the nature of the associations among these parameters warrants further investigation.

## Figures and Tables

**Table 1:** A modified scoring system for evaluation of claw and dew claw lesions, in sows of different genetic lines in three farrow-to-finish herds in Greece.

Region of Interest	Scoring		
	0	1	2
**Claw**	Normal (Size, shape, no anisodactylia)	Mild overgrown and anisodactylia, normally shaped	Overgrown and obvious anisodactylia, abnormal shape
**Wall**	No lesions or very small superficial cracks	Short, vertical or horizontal cracks that do not extend into the corium, a minor separation at the heel–sole and the presence of bruising	Separation of the keratin, a long and severe separation at the heel–sole, deep fractures in the wall extending into the corium or any combination of these
**Coronary band**	No lesions or minor cracks on the surface	Obvious lesions, erosions, swollen edema, hemorrhage and necrosis or both	NA
**Dew claw**	Normal (Size, shape, no lesions)	Slight overgrown without touching the floor and no shape changes	Long with cracks or changes in shape and touching the floor

**Table 2:** Results of maximum temperature (mean ± SD) in each foot at four areas in replacement gilts of different genetic lines in three farrow-to-finish herds in Greece at first farrowing.

	Herds			*p*-Value
	A	B	C	
**IRT1**	*n* = 132	*n* = 84	*n* = 110	
Front right	35.15 ± 1.45	35.58 ± 2.09	35.29 ± 1.85	0.213
Front left	35.12 ± 1.69	35.16 ± 2.29	35.18 ± 1.77	0.969
Rear right	36.05 ± 1.22 ^a^	36.87 ± 1.47 ^b^	36.42 ± 1.46 ^a^	<0.001
Rear left	36.19 ± 1.37 ^a^	36.93 ± 1.24 ^b^	36.44 ± 1.60 ^a^	0.001
**IRT2**				
Front right	34.92 ± 1.57	35.22 ± 2.22	34.95 ± 1.97	0.498
Front left	34.76 ± 1.82	34.72 ± 2.46	34.87 ± 1.84	0.846
Rear right	35.93 ± 1.27 ^a^	36.72 ± 1.52 ^b^	36.11 ± 1.54 ^a^	<0.001
Rear left	32.07 ± 2.68 ^a^	33.69 ± 2.56 ^b^	32.93 ± 2.65 ^b^	<0.001
**IRT3**				
Front right	34.79 ± 1.66	34.73 ± 2.55	34.73 ± 2.08	0.965
Front left	34.55 ± 1.81	34.39 ± 2.63	34.69 ± 2.00	0.601
Rear right	35.65 ± 1.30 ^a^	36.51 ± 1.61 ^b^	35.85 ± 1.62 ^a^	<0.001
Rear left	35.66 ± 1.43 ^a^	35.61 ± 1.41 ^b^	35.90 ± 1.76 ^a^	<0.001
**IRT4**				
Front right	35.60 ± 1.81	35.10 ± 2.82	35.39 ± 2.34	0.298
Front left	35.64 ± 1.88 ^a^	34.86 ± 2.85 ^b^	35.25 ± 2.31 ^a,b^	0.049
Rear right	36.05 ± 1.37	36.52 ± 1.70	36.34 ± 1.53	0.078
Rear left	36.05 ± 1.53 ^a^	36.68 ± 1.52 ^b^	36.35 ± 1.81 ^a,b^	0.021

IRT1: temperature at the carpus in front or tarsus in rear legs; IRT2: temperature at the upper metacarpus or metatarsus area; IRT3: temperature at the lower metacarpus or metatarsus area; IRT4: temperature at the phalanges of each foot. ^a,b^ with a different superscript letter, the means in the same row differ significantly (*p* < 0.05).

**Table 3:** Results of maximum temperature (mean ± SD) in each foot at four areas in replacement gilts of different genetic lines in three farrow-to-finish herds in Greece at second farrowing.

	Herds			*p*-Value
	A	B	C	
**IRT1**	*n* = 55	*n* = 68	*n* = 47	
Front right	35.14 ± 1.59 ^a^	33.76 ± 2.44 ^b^	35.02 ± 2.43 ^a^	<0.001
Front left	35.05 ± 1.43 ^a^	33.55 ± 2.51 ^b^	34.84 ± 2.40 ^a^	<0.001
Rear right	36.49 ± 1.08 ^a^	35.64 ± 1.54 ^b^	36.59 ± 1.38 ^a^	<0.001
Rear left	36.38 ± 1.10 ^a^	35.75 ± 1.52 ^b^	36.43 ± 1.27 ^a^	0.005
**IRT2**				
Front right	34.87 ± 1.57 ^a^	33.38 ± 2.70 ^b^	34.64 ± 2.51 ^a^	0.001
Front left	34.68 ± 1.56 ^a^	33.17 ± 2.66 ^b^	34.48 ± 2.38 ^a^	<0.001
Rear right	36.30 ± 1.08 ^a^	35.41 ± 1.58 ^b^	36.40 ± 1.50 ^a^	<0.001
Rear left	36.22 ± 1.16 ^a^	35.48 ± 1.58 ^b^	36.26 ± 1.34 ^a^	0.001
**IRT3**				
Front right	34.80 ± 1.79 ^a^	33.22 ± 2.61 ^b^	34.59 ± 2.49 ^a^	<0.001
Front left	34.48 ± 1.67 ^a^	32.95 ± 2.73 ^b^	34.36 ± 2.39 ^a^	<0.001
Rear right	36.01 ± 1.10 ^a^	35.09 ± 1.63 ^b^	36.18 ± 1.56 ^a^	<0.001
Rear left	35.93 ± 1.22 ^a^	35.23 ± 1.68 ^b^	36.05 ± 1.45 ^a^	0.003
**IRT4**				
Front right	35.58 ± 1.86 ^a^	33.86 ± 2.54 ^b^	35.25 ± 2.68 ^a^	<0.001
Front left	35.49 ± 1.90 ^a^	33.81 ± 2.60 ^b^	35.11 ± 2.77 ^a^	<0.001
Rear right	36.48 ± 1.34 ^a^	35.30 ± 1.71 ^b^	36.41 ± 1.70 ^a^	<0.001
Rear left	36.44 ± 1.20 ^a^	35.46 ± 1.73 ^b^	36.42 ± 1.61 ^a^	<0.001

IRT1: temperature at the carpus in front or tarsus in rear legs; IRT2: temperature at the upper metacarpus or metatarsus area; IRT3: temperature at the lower metacarpus or metatarsus area; IRT4: temperature at the phalanges of each foot. ^a,b^ with a different superscript letter, the means in the same row differ significantly (*p* < 0.05).

**Table 4:** Results of dorsal claw length (mm) (mean ± SD) at weaning and at first and second farrowing in replacement gilts of three different genetic lines in three farrow-to-finish herds in Greece.

	Herd			*p*-Value
	A	B	C	
Weaning				
**Claws**	*n* = 137	*n* = 98	*n* = 114	
Front Right Medial	18.86 ± 2.24 ^c^	15.07 ± 2.05 ^a^	16.29 ± 1.72 ^b^	<0.001
Front Right Lateral	18.98 ± 2.00 ^c^	14.96 ± 2.00 ^a^	16.33 ± 1.71 ^b^	<0.001
Front Left Medial	18.92 ± 1.97 ^c^	14.92 ± 1.97 ^a^	16.30 ± 1.98 ^b^	<0.001
Front Left Lateral	18.68 ± 1.90 ^c^	15.16 ± 1.86 ^a^	16.33 ± 2.00 ^b^	<0.001
Rear Right Medial	19.40 ± 2.37 ^c^	14.95 ± 2.04 ^a^	16.68 ± 1.94 ^b^	<0.001
Rear Right Lateral	19.47 ± 2.45 ^c^	15.02 ± 1.99 ^a^	16.38 ± 1.76 ^b^	<0.001
Rear Left Medial	19.41 ± 2.29 ^c^	15.19 ± 2.00 ^a^	16.67 ± 1.71 ^b^	<0.001
Rear Left Lateral	19.30 ± 2.42 ^c^	15.22 ± 1.92 ^a^	16.33 ± 1.85 ^b^	<0.001
First Farrowing				
	*n* = 132	*n* = 84	*n* = 110	
Front Right Medial	57.36 ± 8.40 ^b^	56.40 ± 7.35 ^b^	45.93 ± 7.90 ^a^	<0.001
Front Right Lateral	57.96 ± 9.08 ^b^	56.69 ± 9.08 ^b^	46.86 ± 8.05 ^a^	<0.001
Front Left Medial	56.00 ± 8.81 ^b^	55.51 ± 6.69 ^b^	47.55 ± 7.96 ^a^	<0.001
Front Left Lateral	58.10 ± 9.13 ^b^	56.12 ± 8.60 ^b^	47.29 ± 8.58 ^a^	<0.001
Rear Right Medial	60.37 ± 9.44 ^b^	57.82 ± 7.13 ^b^	47.53 ± 8.01 ^a^	<0.001
Rear Right Lateral	63.49 ± 12.93 ^b^	60.74 ± 7.90 ^b^	47.41 ± 7.98 ^a^	<0.001
Rear Left Medial	59.71 ± 9.33 ^b^	57.14 ± 6.75 ^b^	47.58 ± 8.24 ^a^	<0.001
Rear Left Lateral	66.29 ± 12.99 ^c^	62.70 ± 7.87 ^b^	47.00 ± 8.04 ^a^	<0.001
Second Farrowing				
	*n* = 55	*n* = 68	*n* = 47	
Front Right Medial	62.86 ± 12.31 ^b^	59.76 ± 8.84 ^b^	50.84 ± 3.66 ^a^	<0.001
Front Right Lateral	63.36 ± 14.80 ^b^	61.40 ± 8.46 ^b^	48.47 ± 10.18 ^a^	<0.001
Front Left Medial	60.69 ± 12.89 ^b^	59.73 ± 8.06 ^b^	52.25 ± 4.71 ^a^	<0.001
Front Left Lateral	66.22 ± 15.12 ^b^	61.15 ± 9.62 ^b^	50.87 ± 7.82 ^a^	<0.001
Rear Right Medial	70.70 ± 15.55 ^b^	65.81 ± 12.25 ^b^	51.43 ± 4.73 ^a^	<0.001
Rear Right Lateral	73.09 ± 18.95 ^b^	70.84 ± 14.20 ^b^	51.63 ± 4.11 ^a^	<0.001
Rear Left Medial	67.69 ± 14.39 ^b^	65.18 ± 12.34 ^b^	52.22 ± 5.64 ^a^	<0.001
Rear Left Lateral	77.54 ± 18.11 ^c^	69.91 ± 14.67 ^b^	49.56 ± 7.98 ^a^	<0.001

^a–c^ with a different superscript letter, the means in the same row differ significantly (*p* < 0.05).

**Table 5:** Results of anisodactylia (mm) (mean ± SD) in replacement gilts of different genetic lines in three farrow-to-finish herds in Greece, at weaning age and at first and second farrowing.

Foot	Herd			*p*-Value
	A	B	C	
**Weaning**	*n* = 137	*n* = 98	*n* = 114	
Front Right	0.64 ± 0.78 ^a^	0.92 ± 0.81 ^b^	0.87 ± 0.70 ^a,b^	0.009
Front Left	0.66 ± 0.77 ^a^	1.01 ± 0.83 ^b^	0.87 ± 0.71 ^a,b^	0.002
Rear Right	0.56 ± 0.67 ^a^	0.95 ± 0.76 ^b^	0.95 ± 0.71 ^b^	<0.001
Rear Left	0.61 ± 0.92 ^a^	1.02 ± 0.78 ^b^	0.93 ± 0.72 ^b^	<0.001
**First farrowing**	*n* = 132	*n* = 84	*n* = 110	
Front Right	3.32 ± 2.86 ^a^	5.10 ± 6.32 ^b^	2.46 ± 1.91 ^a^	<0.001
Front Left	3.99 ± 4.17 ^b^	4.53 ± 3.80 ^b^	2.77 ± 2.35 ^a^	0.002
Rear Right	9.14 ± 6.35 ^c^	5.89 ± 4.76 ^b^	3.10 ± 2.60 ^a^	<0.001
Rear Left	9.48 ± 6.77 ^c^	6.69 ± 4.99 ^b^	3.33 ± 3.83 ^a^	<0.001
**Second farrowing**	*n* = 55	*n* = 68	*n* = 47	
Front Right	4.90 ± 5.20	3.67 ± 3.12	4.45 ± 9.33	0.526
Front Left	6.93 ± 7.85 ^a^	4.70 ± 3.79 ^a,b^	3.04 ± 6.83 ^b^	0.007
Rear Right	11.09 ± 10.71 ^b^	8.09 ± 6.30 ^b^	2.20 ± 1.93 ^a^	<0.001
Rear Left	13.93 ± 13.37 ^c^	9.09 ± 8.52 ^b^	4.06 ± 6.97 ^a^	<0.001

^a–c^ with a different superscript letter, the means in the same row differ significantly (*p* < 0.05).

**Table 6:** Results of claw lesions (mean ± SD) in replacement gilts of different genetic lines in three farrow-to-finish herds in Greece, at first and second farrowing.

	Herds			*p*-Value
	A	B	C	
**First farrowing**	*n* = 132	*n* = 84	*n* = 110	
Total feet lesions	15.42 ± 11.41 ^a^	11.80 ± 8.47 ^b^	7.62 ± 6.25 ^c^	<0.001
Front feet lesions	7.65 ± 5.27 ^a^	4.65 ± 4.35 ^b^	3.45 ± 3.42 ^b^	<0.001
Rear feet lesions	9.98 ± 6.22 ^a^	7.14 ± 5.33 ^b^	4.24 ± 3.86 ^c^	<0.001
Medial claw lesions	8.34 ± 5.21 ^a^	5.56 ± 4.14 ^b^	3.37 ± 2.92 ^c^	<0.001
Lateral claw lesions	9.30 ± 5.34 ^a^	6.24 ± 4.54 ^b^	4.32 ± 3.45 ^c^	<0.001
**Second farrowing**	*n* = 55	*n* = 68	*n* = 47	
Total feet lesions	17.74 ± 10.23 ^a^	15.48 ± 12.85 ^a^	4.99 ± 6.71 ^b^	<0.001
Front feet lesions	7.76 ± 5.04 ^a^	9.10 ± 5.30 ^a^	3.47 ± 3.05 ^b^	<0.001
Rear feet lesions	9.98 ± 6.24 ^b^	12.22 ± 5.43 ^a^	5.77 ± 4.77 ^c^	<0.001
Medial claw lesions	8.48 ± 5.09 ^a^	9.96 ± 5.44 ^a^	4.09 ± 3.48 ^b^	<0.001
Lateral claw lesions	9.26 ± 5.44 ^b^	11.36 ± 4.96 ^a^	5.15 ± 3.46 ^c^	<0.001

^a–c^ with a different superscript letter, the means in the same row differ significantly (*p* < 0.05).

**Table 7:** Results of backfat thickness (mm), backfat loss (mm) and backfat loss % (mean ± SD) of replacement gilts of different genetic lines in three farrow-to-finish herds in Greece, at farrowing and weaning, and at first and second farrowing.

	Herds			*p*-Value
	A	B	C	
**First farrowing**	*n* = 132	*n* = 84	*n* = 110	
Backfat thickness at farrowing	15.42 ± 2.24 ^b^	20.45 ± 3.34 ^a^	15.30 ± 1.11 ^b^	0.008
Backfat thickness at weaning	12.24 ± 2.39 ^b^	13.86 ± 2.46 ^a^	12.55 ± 1.35 ^b^	<0.001
Backfat Loss	3.19 ± 1.57 ^b^	6.59 ± 2.04 ^a^	2.74 ± 0.99 ^a^	<0.001
Backfat Loss %	20.67 ± 9.78 ^b^	32.02 ± 7.54 ^c^	17.81 ± 5.99 ^a^	<0.001
**Second farrowing**	*n* = 55	*n* = 68	*n* = 47	
Backfat thickness at farrowing	15.09 ± 2.26 ^a^	19.18 ± 2.54 ^b^	14.93 ± 0.96 ^a^	<0.001
Backfat thickness at weaning	12.06 ± 2.27 ^a^	13.20 ± 2.01 ^b^	12.54 ± 0.78 ^a,b^	<0.001
Backfat Loss	3.07 ± 1.55 ^a^	6.11 ± 2.32 ^b^	2.39 ± 0.80 ^a^	<0.001
Backfat Loss %	20.21 ± 9.77 ^b^	31.15 ± 10.17 ^a^	15.87 ± 4.85 ^c^	<0.001

^a–c^ with a different superscript letter, the means in the same row differ significantly (*p* < 0.05).

**Table 8:** Results of mobility score (mean ± SD) of replacement gilts of different genetic lines in three farrow-to-finish herds in Greece, at first and second farrowing.

	Herds			*p*-Value
	A	B	C	
**First farrowing**	*n* = 132	*n* = 84	*n* = 110	
Mobility score	2.19 ± 0.59 ^a^	1.92 ± 0.54 ^b^	1.72 ± 0.74 ^c^	<0.001
**Second farrowing**	*n* = 55	*n* = 68	*n* = 47	
Mobility score	2.66 ± 0.48 ^a^	2.41 ± 0.53 ^b^	2.28 ± 0.58 ^b^	0.002

^a–c^ with a different superscript letter, the means in the same row differ significantly (*p* < 0.05).

**Table 9:** Average values of total born, born alive and weaned piglets at first and second farrowing in sows of different genetic lines in three farrow-to-finish herds in Greece.

	Herds			*p*-Value
	A	B	C	
**First Farrowing**	*n* = 132	*n* = 84	*n* = 110	
Total born	13.86 ± 3.01 ^b^	16.92 ± 3.57 ^a^	13.98 ± 1.64 ^b^	<0.001
Born alive	13.77 ± 3.04 ^b^	15.00 ± 3.17 ^a^	12.27 ± 1.15 ^c^	<0.001
Weaned piglets	12.00 ± 0.00 ^b^	14.57 ± 3.95 ^a^	12.37 ± 2.11 ^b^	<0.001
**Second Farrowing**	*n* = 55	*n* = 68	*n* = 47	
Total born	13.82 ± 3.77 ^b^	17.62 ± 5.20 ^a^	13.57 ± 2.16 ^b^	<0.001
Born alive	13.49 ± 4.01 ^b^	16.20 ± 4.66 ^a^	12.25 ± 1.63 ^b^	<0.001
Weaned piglets	12.00 ± 0.00 ^b^	13.59 ± 2.99 ^a^	11.99 ± 0.83 ^b^	<0.001

^a–c^ with a different superscript letter, the means in the same row differ significantly (*p* < 0.05).

## Data Availability

Data are available from the corresponding author upon request.
